# A general method to determine twinning elements

**DOI:** 10.1107/S0021889810037180

**Published:** 2010-11-09

**Authors:** Yudong Zhang, Zongbin Li, Claude Esling, Jacques Muller, Xiang Zhao, Liang Zuo

**Affiliations:** a Key Laboratory for Anisotropy and Texture of Materials (Ministry of Education), Northeastern University, Shenyang 110819, People’s Republic of China; b Laboratoire d’Étude des Textures et Application aux Matériaux (LETAM), CNRS FRE 3143, Université Paul Verlaine – Metz, Metz 57012, France

**Keywords:** twinning, minimum shear, interface structure, transmission electron microscopy, scanning electron microscopy/electron backscatter diffraction

## Abstract

The fundamental theory of crystal twinning has been long established, leading to a significant advance in understanding the nature of this physical phenomenon. However, there remains a substantial gap between the elaborate theory and the practical determination of twinning elements. This paper proposes a direct and simple method – valid for any crystal structure and based on the minimum shear criterion – to calculate various twinning elements from the experimentally determined twinning plane for Type I twins or the twinning direction for Type II twins. Without additional efforts, it is generally applicable to identify and predict possible twinning modes occurring in a variety of crystalline solids. Therefore, the present method is a promising tool to characterize twinning elements, especially for those materials with complex crystal structure.

## Abstract 2

Based on the minimum shear criterion, a direct and simple method is proposed to calculate twinning elements from the experimentally determined twinning plane for Type I twins or the twinning direction for Type II twins. It is generic and applicable to any crystal structure.

## 1. Introduction

Crystal twins are commonly observed during solidification, deformation, solid-state phase transformation and recrystallization in a variety of crystalline solids with low stacking fault energy. Often, these features occur on the nanometre to micrometre scale, and they represent a particularly symmetric kind of grain boundary, giving rise to a much lower level of interfacial energy than general grain boundaries. As an underlying mechanism for microstructural changes, crystal twinning has acquired great importance in fields such as metallography, mineralogy, crystallography and physics.

Early efforts to define crystal twins were based on the study of deformation twinning. By convention, a deformation twin is a region of a crystal that has undergone a homogeneous shape deformation (simple shear) in such a way that the resulting structure is identical to that of the parent (matrix), but differently oriented. A twinning mode is fully characterized by six elements: (1) *K*_1_ – the twinning or composition plane that is the invariant (unrotated and undistorted) plane of the simple shear; (2) η_1_ – the twinning direction or the direction of shear lying in *K*_1_; (3) *K*_2_ – the reciprocal or conjugate twinning plane, the second undistorted but rotated plane of the simple shear; (4) η_2_ – the reciprocal or conjugate twinning direction lying in *K*_2_; (5) *P* – the plane of shear that is perpendicular to *K*_1_ and *K*_2_ and intersects *K*_1_ and *K*_2_ in the directions η_1_ and η_2_, respectively; (6) γ – the magnitude of shear. Moreover, the orientation relationship between two twin-related crystals can be specified by simple crystallographic operations: a reflection across *K*_1_ or a 180° rotation about the direction normal to *K*_1_; or a 180° rotation about η_1_ or a reflection across the plane normal to η_1_. According to the rationality of the Miller indices of *K*_1_, *K*_2_, η_1_ and η_2_ with respect to the parent lattice, crystal twins are usually classified into three categories: Type I twin (*K*_1_ and η_2_ are rational), Type II twin (*K*_2_ and η_1_ are rational) and compound twin (*K*_1_, *K*_2_, η_1_ and η_2_ are all rational).

The classical definition and description of deformation twinning have been further extended to describe other twinning processes associated with phase transformation and recrystallization. Notably, the concept of transformation twinning is widely adopted for the elucidation of structural changes during martensitic transformation. Although the formation of twinned martensitic variants is driven by a deformation from the parent phase and may not have any relation to the simple shear deformation defined by the twinning shear, the detwinning process can be well predicted by these elements, especially for the newly developed ferromagnetic shape memory alloys (Gaitzsch *et al.*, 2009 ▶; Wang *et al.*, 2006 ▶; Li *et al.*, 2010 ▶). In such a case, the twinned martensitic variants always form regular arrays of alternate lamellae with fixed thickness and the twin boundaries are highly glissile, where the detwinning shear determines the shape memory performance.

For many years, constant attempts have been made to determine twinning elements of crystalline materials from the knowledge of crystal structure, because of their importance for insight into possible twinning modes and resultant orientation relationships of twinned crystals in the context of microstructural manipulation. A systematic theory was developed by Kiho (1954 ▶, 1958 ▶) and Jaswon & Dove (1956 ▶, 1957 ▶, 1960 ▶) based on the minimum shear criterion, and later completed by Bilby & Crocker (1965 ▶) and Bevis & Crocker (1968 ▶, 1969 ▶). It provides the general expressions – valid for all crystal structures – to predict the twinning elements for both Type I and Type II twins with a known twinning shear. However, in a practical determination of unknown twins, it is only feasible to resolve the possible twinning plane *K*_1_ for Type I twins or the twinning direction η_1_ for Type II twins by means of transmission electron microscopy (TEM) or scanning electron microscopy/electron backscatter diffraction (SEM/EBSD). In other words, with the given general expressions, one always suffers from insufficient information to derive the unknown twinning elements, especially the twinning shear. As a common practice, laborious geometrical examination of the lattice correspondence of the stacking planes parallel to the twinning plane has to be conducted. Such a process becomes particularly difficult when the twinning plane and the shear plane are irrational and the crystal structure is complicated. Hence, there exists a substantial gap between the elaborate theory and the practical determination.

In this paper, we present a complete method to find all twinning elements for the three classical types of twins, based on the assumption that a simple minimum shear operation transforms the lattice points of a crystal into their counterpart twin positions. The initial inputs are simply the crystal structure and the experimentally determined *K*_1_ (Type I) or η_1_ (Type II). As a general method applicable to any crystal structure, it may facilitate future characterization studies of crystal twinning.

## 2. Methodology

### 2.1. Determination of twinning mode

For a twinned crystal, the crystallographic orientations of the twin and its parent can be experimentally determined with SEM/EBSD or TEM. In the case of SEM/EBSD examination, the orientation of a crystal with respect to the macroscopic sample coordinate system is usually characterized in terms of three Euler angles. The misorientation between the twin and the parent is then calculated from their Euler angles, and expressed by a set of rotation angles and the corresponding rotation axes (Cong *et al.*, 2006 ▶, 2007 ▶). According to the definition of twin relationships mentioned above, there exists at least one 180° rotation. If the Miller indices of the plane normal to the 180° rotation axis are rational, the twinning mode belongs to Type I and the plane is the twinning plane *K*_1_. If the Miller indices of the 180° rotation axis are rational, the twinning mode refers to Type II and the direction of the rotation axis is the twinning direction η_1_. Since a compound twin has two 180° rotations with rational *K*_1_, *K*_2_, η_1_ and η_2_, the plane normal to the 180° rotation axis that offers the minimum shear should be the twinning plane *K*_1_.

In contrast to the SEM/EBSD examination, the TEM determination process involves examining the spot diffraction image (Nishida *et al.*, 2008 ▶). For Type I and compound twins, the diffraction image – obtained on condition that the incident beam is parallel to the *K*_1_ plane – consists of two sets of reflections that are in mirror symmetry to each other with respect to the *K*_1_ reflection. Thus, the *K*_1_ plane can be identified. For Type II twins, the diffraction image – obtained with the incident beam along the η_1_ direction – contains a single visible pattern, *i.e.* the reflections from two twin-related crystals overlap each other. The η_1_ direction could also be determined.

Based on the above experimental identification, the other twinning elements to define a twinning mode can be further derived with the method outlined below.

### 2.2. Determination of twinning elements

#### 2.2.1. Type I and compound twins

According to the classical definition, a Type I or compound twin is related to its parent by a reflection across the twinning plane *K*_1_, where the *K*_1_ plane is a rational lattice plane with relatively small Miller indices. With this condition as starting point, the possible twinning direction η_1_ and the magnitude of twinning shear γ can be deduced in conformity with the minimum shear criterion, *i.e.* the twinning shear that moves all parent lattice points to their correct twin positions appears to be the smallest in magnitude. Hereafter, our calculations are conducted in the direct primitive lattice of the parent crystal. For the coordinate transformations between the primitive lattice basis and the conventional Bravais lattice basis, we refer to *International Tables for Crystallography* (Hahn, 1996 ▶).

**Figure 1:**
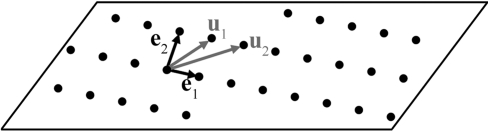
Lattice plane *K*_1_ with basis vectors **u**_1_ and **u**_2_ and reduced basis vectors **e**_1_ and **e**_2_.

At first, let us choose two basis vectors **u**_1_ and **u**_2_ in the twinning plane *K*_1_ and transform them into the reduced vectors **e**_1_ and **e**_2_, as shown schematically in Fig. 1 ▶. The reduced basis vectors **e**_1_ and **e**_2_ must be the two shortest translations and the most orthogonal to each other among all possible basis vectors in the plane *K*_1_. Note that such a reduced basis is useful for determining the nearest lattice point(s) to a given point (not necessarily lattice site) in the plane *K*_1_. The procedures to find the basis vectors **u**_1_ and **u**_2_ and to reduce them to **e**_1_ and **e**_2_ are detailed in Appendix *A* [App appa] and Appendix *C* [App appc], respectively.

**Figure 2:**
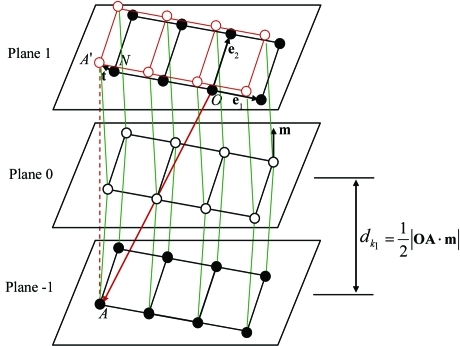
Illustration of the nearest neighbor plane (Plane −1) of the parent lattice and its counterpart (Plane 1) for the twin lattice, which are parallel and in mirror symmetry with respect to the invariant plane *K*_1_ (Plane 0). The twinning shear vector **t** is represented by the displacement from a parent lattice point *N* to the nearest twin lattice position *A*′ on Plane 1.

Now, we show how to determine the twinning shear vector **t** by use of the reduced basis **e**_1_ and **e**_2_. Let Plane 0 represent the twinning (invariant) plane *K*_1_ that separates the twin lattice (above Plane 0) from that of the parent (below Plane 0), as shown schematically in Fig. 2 ▶. Since the nearest neighbor plane (Plane −1) of the parent lattice and its counterpart (Plane 1) for the twin lattice are parallel and in mirror symmetry with respect to the invariant plane *K*_1_, the perpendicular projection of Plane −1 onto Plane 1 allows us to identify the possible twinning shear vector. Here, we select a parent lattice vector **OA** that ends at the lattice point *A* on Plane −1, and denote by *A*′ the endpoint of the projection of vector **OA** on Plane 1. Obviously, the vector **t** that joins *A*′ – a twin lattice point – to its nearest parent lattice point *N* on Plane 1 defines the twinning direction η_1_ and ensures the smallest magnitude of shear. The procedures for determining the vectors **OA** and **t** are described in Appendix *B* [App appb].

Furthermore, the interplanar spacing of the twinning plane *K*_1_ can be easily calculated by the scalar product of **OA** and **m**: 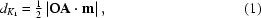
where **m** denotes the unit vector in the direction normal to the twinning plane *K*_1_. Thus, the magnitude of shear is given by 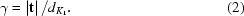
Once the shear vector **t** and the magnitude of shear γ are determined, the other twinning elements (η_2_, *K*_2_ and *P*) can be readily calculated according to the Bilby–Crocker theory (Bilby & Crocker, 1965 ▶).

**Figure 3:**
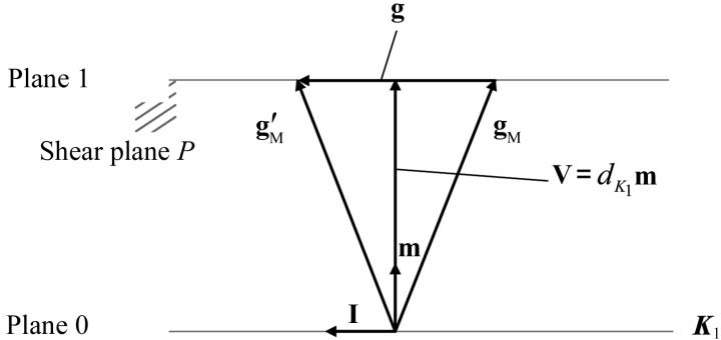
Transformation of vector **g**_M_ (in the direction η_2_) into vector **g**_M_′ by a magnitude of shear γ along the direction η_1_. Note that **g**_M_ and **g**_M_′ have the same length and are in mirror symmetry with respect to the plane perpendicular to the *K*_1_ plane and the shear plane *P*.

Let **I** be the unit vector in the twinning direction η_1_ and **g**_M_ a vector in the conjugate twinning direction η_2_, with reference to the parent lattice basis. Applying the twinning operation by a shear γ along η_1_, **g**_M_ is transformed into **g**_M_′, as shown schematically in Fig. 3 ▶. Since η_2_ is defined by a rotated but undistorted lattice line of the shear, **g**_M_′ has the same indices (and hence the same length) as **g**_M_, if it is referred to the twin lattice basis. Moreover, **g**_M_ and **g**_M_′ lying in the shear plane *P* (perpendicular to *K*_1_) are in mirror symmetry with respect to the plane that contains the vector **V** (= 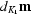) and is perpendicular to η_1_. Thus, the three vectors **g**_M_, **g**_M_′ and **g** form an isosceles triangle. As **g** (= 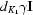) in the shear direction is divided into two equal lengths by **V**, we obtain 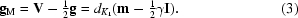

               

Notably, **g**_M_ is not necessarily a lattice vector, and its components – expressed in terms of the parent lattice basis – can always be transformed into rational indices. Once the lattice vector in the η_2_ direction is determined from **g**_M_, the shear plane *P* and the conjugate twinning plane *K*_2_ can be easily calculated by the vector cross product (Bilby & Crocker, 1965 ▶).

#### 2.2.2. Type II twin

By definition, a Type II twin is related to its parent by a 180° rotation about the twinning direction η_1_ or a reflection across the plane normal to the twinning direction η_1_. Let us first recall the fundamental relationships between direct lattice and reciprocal lattice. Every lattice vector in the direct space corresponds to a set of lattice planes normal to this vector in the reciprocal space, and *vice versa*. Thus, the twin relationship of a Type II twin in the direct space can be equivalently expressed by a reflection with respect to the plane that is normal to η_1_ in the reciprocal space, or in other words, a Type II twin in the direct space is visualized as a Type I twin in the reciprocal space. As the two spaces are strictly linked to each other, we can see that, when the direct lattice undergoes twinning, the reciprocal lattice is subject to the same deformation (shear in the same direction and with the same magnitude) and *verse visa*. In this context, the determination of the twinning elements of Type II twins can follow the same procedure as that of Type I, except that all the calculations should be conducted in the reciprocal space. Moreover, the resultant directions (planes) in the reciprocal space correspond to the same indexed planes (directions) in the direct space, as summarized in Table 1 ▶.

## 3. Conclusions

As a widely observed and intrinsic process, crystal twinning has a broad impact on the microstructures and properties of crystalline materials. So far, the classical theory of twinning has advanced greatly the study of twining, but it often suffers from insufficient information for practical determination of full twinning elements. To progress beyond this state, a general method is elaborated based on the minimum shear criterion, using the experimentally identified possible twinning plane *K*_1_ for Type I twins or the twinning direction η_1_ for Type II twins and the crystal structure as input. As a first step, it determines a reduced basis of the invariant lattice plane that serves as the mirror plane (in the direct space for Type I twins and in the reciprocal space for Type II twins) between the parent and twin lattices. Then, a lattice vector – with its origin at the invariant lattice plane and its end at the nearest neighbor lattice plane of the same set – is selected from the parent lattice and projected onto the counterpart lattice plane of the twin lattice. Among the vectors that join the endpoint of the projected lattice vector to the surrounding parent lattice points forming the reduced basis, the shortest vector defines the twinning direction and the twinning shear. Finally, the other twinning elements can be easily calculated using the vector product operations. The present method, as it stands, is highly significant for facilitating the study of twinning in a variety of crystalline materials.

## Figures and Tables

**Figure 4:**
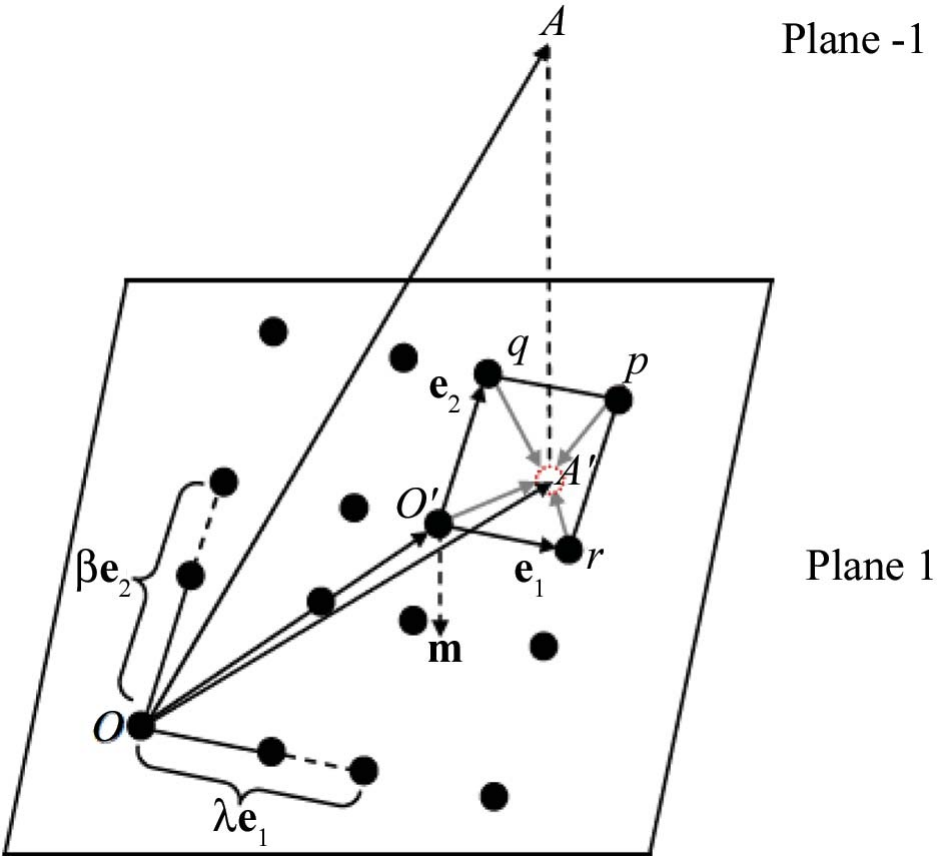
Perpendicular projection *A*′ of a lattice point *A* of Plane −1 onto Plane 1. The shortest vector joining *A*′ to the nearest lattice point is denoted as the shear vector.

**Table 1:** Reciprocal relationship of twinning elements in dual spaces

Direct space	Reciprocal space
*K*_1_	η_1_
η_1_	*K*_1_
*K*_2_	η_ 2_
η_2_	*K*_2_
*P*	Normal to *P*
γ	γ

## Appendix A Determination of base vectors **u**_1_ and **u**_2_ on a lattice plane

In crystallography, a lattice plane *P* with a given Bravais lattice is usually described by the Miller indices (*hkl*), *i.e.* a set of three integers with the greatest common divisor  . Assume ; then . If an arbitrary lattice vector **u** with the Miller indices [*uvw*] lies in the plane *P*, it has

where *u*, *v* and *w* are integers. Since , the following relation holds:

Let ,  and ; then ,  and  are also integers. Equation (5) can be written as

As , one can find two integers  and  that satisfy the following relation according to Bézout’s theorem:

Multiplying both sides of equation (7) by (), we obtain

Let  and ; then equation (8) becomes

Subtracting equation (9) from equation (6), we have

Since , there exists an integer  such that

Rearranging equation (11), we obtain

By definition, the basis vectors are a set of linearly independent vectors such that each vector in the space is a linear combination of the vectors from the set. Therefore, equation (12) proves that the vector (, , ) constitutes the basis vectors of the plane (*hkl*). Setting  and , and  and , respectively, we obtain two basis vectors:

where  and  are the Bézout coefficients of equation (7). With the Euclidean algorithm,  and  can be easily calculated.

## Appendix B Determination of lattice vector **OA** and shear vector **t**

### b1. Lattice vector **OA**

According to the fundamental law of the reciprocal lattice (Authier, 2001 ▶), for an arbitrary vector **OA** with its origin *O* at the zeroth plane of a family of lattice planes (*hkl*), if it intersects the *n*th plane at the point with coordinates (*x*, *y*, *z*), the following relation holds:

Let **OA** be the lattice vector with the Miller indices [−2*u* −2*v* −2*w*] and *K*_1_ the invariant plane with the Miller indices (*hkl*), as shown in Fig. 2 ▶. Then, we have

The Bézout coefficients *u*_0_, *v*_0_ and *w*_0_ of equation (15) can be calculated with the Euclidean algorithm, and hence the lattice vector **OA**.

### b2. Shear vector **t**

Consider a lattice vector **OA** with its origin at *O* on Plane 1 and its end at *A* on Plane −1, as shown in Fig. 4 ▶.

Let *A*′ be the perpendicular projection of the lattice point *A* on Plane 1. The shear vector **t** is defined as the shortest vector among all vectors that connect *A*′ with the surrounding lattice points on Plane 1. Introducing the reduced basis **e**_1_ and **e**_2_, we can derive from Fig. 4 ▶ that

where **m** is the unit vector of the plane normal. By comparing the lengths of **O**′**A**′, **qA**′, **pA**′ and **rA**′, the shortest vector **t** can be easily found.

## Appendix C Transformation of basis vectors **u**_1_ and **u**_2_ into the reduced basis **e**_1_ and **e**_2_

To find the closest lattice point to the projection *A*′ and thus the minimum shear of the twinning, it is essential to establish a reduced basis, *i.e.* the two shortest lattice vectors that are most orthogonal to each other (Zuo *et al.*, 1995 ▶). With the basis vectors **u**_1_ and **u**_2_ determined according to Appendix *A* [App appa] as input, the reduced basis **e**_1_ and **e**_2_ can be derived using an iterative procedure, as described below.

Let **e**_1_ be the shorter vector between the two base vectors, *i.e.* . Then, the new base vectors are derived from

To render the two vectors orthogonal to each other, this yields

If ,  and  deliver the reduced basis vectors. Otherwise,  is rounded into the nearest integer and the above procedure is repeated until .

## References

[bb1] Authier, A. (2001). *The Reciprocal Lattice.* IUCr Pamphlet Series, No. 4, http://www.iucr.org/education/pamphlets.

[bb2] Bevis, M. & Crocker, A. G. (1968). *Proc. R. Soc. London Ser. A*, **304**, 123–134.

[bb3] Bevis, M. & Crocker, A. G. (1969). *Proc. R. Soc. London Ser. A*, **313**, 509–529.

[bb4] Bilby, B. A. & Crocker, A. G. (1965). *Proc. R. Soc. London Ser. A*, **288**, 240–255.

[bb5] Cong, D. Y., Zhang, Y. D., Wang, Y. D., Esling, C., Zhao, X. & Zuo, L. (2006).* J. Appl. Cryst.* **39**, 723–727.

[bb6] Cong, D. Y., Zhang, Y. D., Wang, Y. D., Humbert, M., Zhao, X., Watanabe, T., Zuo, L. & Esling, C. (2007). *Acta Mater.* **55**, 4731–4740.

[bb7] Gaitzsch, U., Potschke, M., Roth, S., Rellinghaus, B. & Schultz, L. (2009). *Acta Mater.* **57**, 365–370.

[bb8] Hahn, T. (1996). *International Tables for Crystallography*, Vol. A, 4th ed., pp. 76–80. Dordrecht: Kluwer Academic Publishers.

[bb9] Jaswon, M. A. & Dove, D. B. (1956). *Acta Cryst.* **9**, 621–626.

[bb10] Jaswon, M. A. & Dove, D. B. (1957). *Acta Cryst.* **10**, 14–18.

[bb11] Jaswon, M. A. & Dove, D. B. (1960). *Acta Cryst.* **13**, 232–240.

[bb12] Kiho, H. (1954). *J. Phys. Soc. Jpn*, **9**, 739–747.

[bb13] Kiho, H. (1958). *J. Phys. Soc. Jpn*, **13**, 269–272.

[bb14] Li, Z., Zhang, Y., Esling, C., Zhao, X., Wang, Y. & Zuo, L. (2010). *J. Appl. Cryst.* **43**, 617–622. 10.1107/S0021889810037180 PMC3253729 22477779

[bb15] Nishida, M., Hara, T., Matsuda, M. & Ii, S. (2008). *Mater. Sci. Eng. A*, **481–482**, 18–27.

[bb16] Wang, Y. D., Ren, Y., Li, H. Q., Choo, H., Benson, M. L., Brown, D. W., Liaw, P. K., Zuo, L., Wang, G., Brown, D. E. & Alp, E. E. (2006). *Adv. Mater.* **18**, 2392–2396.

[bb17] Zuo, L., Muller, J., Philippe, M.-J. & Esling, C. (1995). *Acta Cryst.* A**51**, 943–945.

